# Some Complications of Ruptured Liver

**Published:** 1908-09

**Authors:** Lionel Shingleton Smith


### SOME COMPLICATIONS OF RUPTURED LIVER.

BY

LIONEL SHINGLETON SMITH, M.B. Cantab.

---

THE liver is liable to very serious injury, which may result in the death of the patient in a few minutes from internal hemorrhage; or if the bleeding is checked, either by the resistance of the liver capsule and ligaments or by surgical means, the patient may survive; or a sub-capsular hæmatoma or sub-phrenic hæmatoma may form, and on the addition of pyogenic organisms, brought thither by the portal blood, an abscess in the liver substance or under the diaphragm is formed, which, if left unopened, must terminate fatally.

Generally, a severely ruptured liver is accompanied by some other lesion, of which the following are commonly found *post-mortem*:—Fractured ribs, lacerated lung, ruptured spleen, ruptured kidney, and ruptured diaphragm, numerous recorded instances of which can be seen in the surgical and *post-mortem* books of the Bristol Royal Infirmary.

With these conditions I am not going to deal, but to touch lightly on those cases which are less severe, and at the time may be even overlooked, but which are liable to terminate fatally as the result.

On September 13th, 1907, a woman, æt. 50, was admitted to the South Devon and East Cornwall Hospital under the care of Mr. Woollcombe, suffering from what appeared to be a typical empyema.

*History.*—The patient had been in good health for the past fifteen years till ten days ago, when she was taken with vomiting and shivering fits. Her friends put this illness down to a blow with a broom-handle which her husband had dealt her some six months before.

*On admission* the patient was feverish, with temperature 102.8°, and had a severe pain in the right side over the lower ribs. A fulness with obliteration of the intercostal spaces was observed, diminished vocal vibration, marked dulness on percussion, and diminished voice and breath sounds were elicited over the lower part of the right chest, behind and in the midaxillary line, but in front the chest was resonant. The exploring needle was inserted in several places behind, but no fluid was detected. *September 24th:* A Turner's trochar was inserted in the ninth interspace, and much thick fluid of a chocolate colour drained away; this continued to drain freely for some days. *October 9th:* The wound was enlarged, and a piece of rib removed, after which more of this pus came away, and thick masses of slough were scraped away with a spoon, and two rubber tubes inserted. *October 19th:* The tubes drained well, and were irrigated through twice daily, but the temperature kept up to 100 and 101° F., when a large, tender inflammatory swelling became apparent in the right groin. This was opened under local anæsthesia, and a tablespoonful of thick white pus let out; the abscess cavity was limited all round, except above, where it seemed to extend well above Poupart's ligament. The patient became gradually worse, the abscess in the groin closed up, and she died on November 25th.

*Autopsy.*—The sinus in the chest was seen to lead down below and behind the liver to the upper surface of that organ. Lungs, normal. The liver was found to contain a large abscess as big as one's fist, occupying the upper and posterior portion of the liver, and separated from the diaphragm by a very thin layer of liver substance. The sinus was traced down to the liver, but there was found no communication between it and the liver abscess. A large psoas abscess was also present, pointing in the right groin; this was traced close up to the hepatic abscess, but no opening could be found leading from one to the other. The appendix was normal, and there was not, nor had been, any inflammation in that region. The pus was white, and the only organisms found were staphylococci. There were no hooklets present to point to hydatid disease.

This patient had never been abroad, and therefore a tropical abscess may be excluded. There were no hooklets to indicate hydatid disease; no intestinal foci of suppuration which might cause secondary hepatic abscesses, and therefore a suppurating hæmatoma was evidently the cause of the illness and death, though the injury at the time was ignored.

Abdominal injuries, resulting in a ruptured liver of greater extent than in the case above mentioned, and which give rise to hemorrhage sufficient to be diagnosed by the pallor, rapid pulse, abdominal tenderness, and nature of the blow received, give an opportunity for removal of the blood clot and suture of the tear in the liver, thus giving the patient a better prospect than in those cases where the blow has passed unnoticed and a hæmatoma resulted, followed by suppuration.

A girl, æt. 14, was brought to the casualty room of the Bristol Royal Infirmary and admitted under Mr. Bush, with the history of having hurt her abdomen whilst playing, by falling across the curb-stone.

*On admission* she was very pale, perfectly conscious, and complained of pain in the umbilical region. *August 13th,* 1906: Temperature 99.2°; abdomen moves on respiration, nothing abnormal felt. *August 14th:* The child looks very ill, and has been vomiting all night. Face flushed, tongue dry, with a brown fur. Abdomen rigid and tender, especially at the upper part of the right rectus muscle. There is no increased dulness to indicate hemorrhage. Pulse 128. *Operation:* The abdomen was incised in the mid line above the umbilicus. In the extra-peritoneal fat were seen one or two spots of a light canary colour, which were thought to be fat necrosis. The omentum was found to be studded with these yellowish-white nodules, but the peritoneal coat of the bowel was free from them. In the right hypochondrium blood was seen free in the peritoneal cavity, and a small rupture of the liver was seen immediately to the right of the notch for the round ligament. There was no apparent rupture of the pancreas, the bleeding had ceased, the blood was removed, the liver rupture sewn up, and the abdomen closed. *August 18th:* On examination of the chest, cardiac pulsation could be felt to the right and left of the sternum, in the fourth and fifth spaces; apex not located. The cardiac dulness extended for one inch to the right and one inch to the left of the sternum in the fifth space, so that the heart has become displaced, and lay mesially behind the sternum. The lower part of the right chest in front yielded a curious tympanitic note on percussion, like that elicited over a pneumothorax or large cavity. Posteriorly, the right base had an impaired note with cavernous breathing, bronchophony, pectoriloquy, and a few crepitations. Over the left side of the chest the normal lung resonance was present, and the air entry was good all over. There was, apparently, nothing present in the left side of the chest to push the heart over to the right, and therefore it must have been pulled over by the changes occurring in the right side of the chest. *August 21st:* Right lung, consolidation of the lower lobe present; some pus expectorated. *August 24th:* Temperature 96.8°. Tubular breathing heard over the right lower lobe. The purulent expectoration still continued, and was accompanied by a little blood. The lung was clearing up, a few coarse crepitations were heard over the right base. *September 1st:* Expectoration had ceased; temperature normal. *September 23rd:* The girl left the Infirmary in perfect health.

In this case the abdominal tenderness and rigidity over the upper part of the right rectus muscle, together with the history of a blow, left little doubt of the diagnosis. At the operation the hemorrhage was found to have stopped, and the liver was sutured up, thus removing the possibility of fresh bleeding when the patient began to recover, and the blood-pressure to rise.

The reason for the fat necrosis is not obvious, but one must suspect that the pancreas was bruised, and that there had been some escape of pancreatic secretion, though, as far as could be seen at the operation, that organ was not ruptured.

The very unusual condition of the chest, which was apparent three days after the operation, could not have been due to any long-standing tubercular disease as was suggested at the time, because the patient went out well. The signs present were evidently those of pneumothorax, and this could only have been brought about by a laceration of the lung through the visceral pleura into a small bronchus; air escaped into the pleural cavity, the intra-pleural pressure was raised, and the lung collapsed. Under such circumstances it is usual to find the heart pushed over to the unaffected side—in this case to the left side of the chest—and I can only suggest that there had been some old standing pleurisy or mediastinitis causing the lung to be bound down to the pleura and pericardium, then when the lung collapsed the pericardium and heart were drawn over to the right.

A collection of blood in contact with air readily suppurates, and thus the blood effused into the lung tissue in connection with the bronchus became purulent, and was got rid of by expectoration.

Therefore, this girl had ruptured her liver and right lung, probably also bruised her pancreas, and yet made a perfect recovery.

